# Remote cardiac rehabilitation is a good alternative of outpatient cardiac rehabilitation in the COVID-19 era

**DOI:** 10.1186/s12199-020-00885-2

**Published:** 2020-09-05

**Authors:** Atsuko Nakayama, Naoko Takayama, Momoko Kobayashi, Kanako Hyodo, Naomi Maeshima, Fujiwara Takayuki, Hiroyuki Morita, Issei Komuro

**Affiliations:** 1 Department of Cardiovascular Medicine, Graduate School of Medicine, The University of Tokyo, 7-3-1 Hongo, Bunkyo-ku, Tokyo, 113-8655 Japan; 2 Nursing Department, The University of Tokyo, Tokyo, Japan

**Keywords:** Cardiac rehabilitation, Remote medicine, COVID-19

## Abstract

**Background:**

In the wake of the coronavirus disease 2019 (COVID-19) pandemic, people need to practice social distancing in order to protect themselves from SARS-CoV-2 infection. In such stressful situations, remote cardiac rehabilitation (CR) might be a viable alternative to the outpatient CR program.

**Methods:**

We prospectively investigated patients hospitalized for heart failure (HF) with a left ventricular ejection fraction of < 50%. As for patients who participated in the remote CR program, telephone support was provided by cardiologists and nurses who specialized in HF every 2 weeks after discharge. The emergency readmission rate within 30 days of discharge was compared among the outpatient CR, remote CR, and non-CR groups, and the EQ-5D score was compared between the outpatient CR and remote CR groups.

**Results:**

The participation rate of HF patients in our remote CR program elevated during the COVID-19 pandemic. As observed in the outpatient CR group (*n* = 69), the emergency readmission rate within 30 days of discharge was lower in the remote CR group (*n* = 30) than in the non-CR group (*n* = 137) (*P* = 0.02). The EQ-5D score was higher in the remote CR group than in the outpatient CR group (*P* = 0.03) 30 days after discharge.

**Conclusions:**

Remote CR is as effective as outpatient CR for improving the short-term prognosis of patients hospitalized for heart failure post-discharge. This suggests that the remote CR program can be provided as a good alternative to the outpatient CR program.

## Background

In the pandemic caused by SARS-CoV-2 infection, people worldwide are trying to prevent the spread of infection by practicing social distancing. Prolonged isolation at home leads to reduced physical activity in the general population, and the risks associated with physical inactivity are elevated, especially in patients with cardiac disease. However, now, many hospitals may not provide outpatient cardiac rehabilitation (CR) services in order to minimize the risk of SARS-CoV-2 infection in hospitals.

In Japan, the first coronavirus disease 2019 (COVID-19) case was reported on January 16, 2020; at that time, most hospitals continued outpatient CR. However, the incidence rate of infection gradually increased, and outpatient CR in Japan required close attention. During this period, we prohibited patients with common cold symptoms such as fever from participating in CR and encouraged all patients to wear masks in the exercise room. We conducted careful outpatient CR, disinfecting the ergometer handles and saddles with alcohol before and after each procedure. These prophylactic methods against COVID-19 for outpatient CR were recently recommended in the guideline prescribed by the European Association of Preventive Cardiology [1]. However, when a COVID-19 case was reported in a sports gym, our hospital stopped providing outpatient CR services on March 4, 2020, a month ahead of the general hospitals since our hospital has a large heart transplantation center, with many patients suffering from severe heart failure (HF). The government declared a state of emergency on April 7 and asked the general public, the healthcare professionals, and medical contributors to refrain from unnecessary hospital visits. Due to this emergency declaration (ED), outpatient CR in most of the facilities has been interrupted. In addition, cardiologists in charge of outpatient CR also had to be involved in the treatment of SARS-CoV-2 infections, resulting in a greater mental and physical burden on them. Therefore, outpatient CR services were postponed.

The rate of readmission of patients with HF increases due to the inability to provide adequate medical care, including outpatient CR service [2]. What kind of CR can we provide in this COVID-19 era? Is it possible to provide safe and effective CR for patients with cardiac disease in their own houses? Here, we demonstrate the effectiveness of our remote CR program which had been launched in 2019, before the beginning of the COVID-19 pandemic.

## Methods

**Fig. 1:**
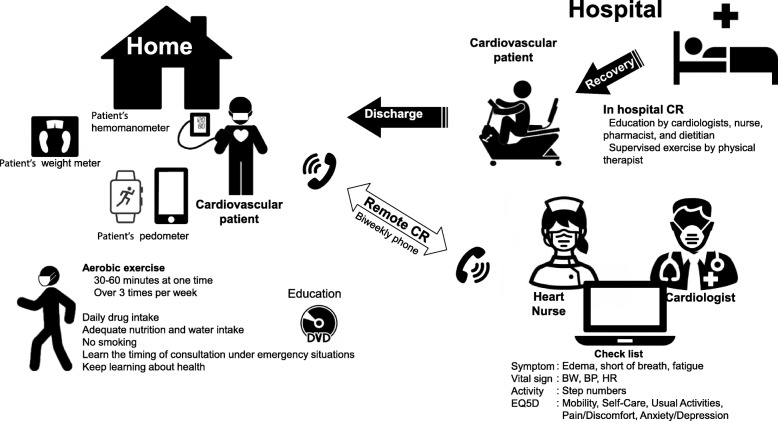
Schema of remote CR supervised by medical staff. Patients who participated in remote CR were given digital video disk (DVD) guides for home CR created by our CR staff; after discharge, telephone consultation services were provided to them by cardiologists and specialized nurses, every 2 weeks for 5 months. CR, cardiac rehabilitation; HF, heart failure; BW, body weight; BP, blood pressure; HR, heart rate

Based on previous reports on successful tele-CR [3–5], we launched a home-based CR program for patients hospitalized for HF with a left ventricular ejection fraction (EF) of < 50%, in January 2019. In the early phase of hospitalization, CR for patients with HF is initiated. CR for severe cases of HF (New York Heart Association [NYHA] class IV) is generally contraindicated. However, it is performed when disuse syndromes can potentially be prevented with careful bedside strength training. At discharge, patients could choose to participate in outpatient CR, remote CR, or non-CR. Outpatient CR was provided as a usual CR program according to the guideline by the Japan Circulation Society [6]. Patients who participated in remote CR were given digital video disk (DVD) guides for home CR, created by our CR staff; after discharge, a telephone consultation service was provided to the patients by cardiologists and specialized nurses, every 2 weeks for 5 months (Fig. 1). In our 15-min home CR film, the CR staff explained heart failure, warming-up exercise, aerobic exercises in and out of home, and possible symptoms requiring emergency visits. During the telephone consultations with the CR staff, patients were asked about the presence of leg edema, shortness of breath, and symptoms of fatigue along with body weight trends, blood pressure trends, pedometer trends, and questions on quality of life (QOL) based on EQ-5D [7].

**Fig. 2:**
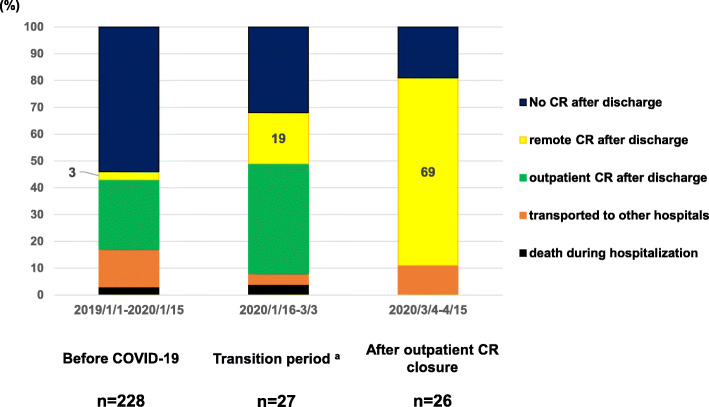
Remote cardiac rehabilitation for patients with heart failure before and during the COVID-19 pandemic. CR after discharge for patients with HF before and during the COVID-19 pandemic. Superscript letter “a” denotes the period from the first report of COVID-19 in Japan to outpatient CR closure in our hospital. CR, cardiac rehabilitation

Before outpatient CR closure in our hospital (on March 4, 2020), patients who were hospitalized for HF could choose whether to participate in outpatient CR, remote CR, or non-CR at discharge (Fig. 2). To reduce the risk of SARS-CoV-2 infection, we promoted the remote CR option for HF patients. The patients who switched from the outpatient CR program to the remote CR program within 30 days of discharge were excluded, and emergency readmission rates within 30 days of discharge in patients with outpatient CR, remote CR, and non-CR were compared using the *χ*^2^ test. The EQ-5D scores in patients with outpatient CR and remote CR were compared using the unpaired *t* test.

This study was based on the principles of the Declaration of Helsinki and approved by the ethical committees of the University of Tokyo Hospital, Tokyo, Japan (approval ID 2650-6).

## Results

The number of participants in the remote CR program increased after the onset of SARS-CoV-2 infections; after March 4, 2020, our hospital stopped providing outpatient CR services, and instead, the remote CR service was provided after discharge. Accordingly, the proportion of the patients who initiated remote CR after discharge dramatically elevated from 19 to 69% (Fig. 2). One patient who participated in the outpatient CR program and switched to the remote CR program within 30 days after discharge was excluded from the analysis. The dropout rate from the remote CR program was 0%.

In a total of 236 patients hospitalized for HF (Table 1), there was a higher proportion of elderly and female patients in the remote CR and non-CR groups as compared to the outpatient CR group (70 ± 9 years, 69 ± 20 years, and 59 ± 18 years, *P* = 0.01; 60%, 59%, and 39%, *P* = 0.02, respectively). The rate of emergency readmission within 30 days of discharge was lower in the CR groups, including the outpatient CR (2 out of 69, 3%) and remote CR (0 out of 30, 0%) groups, as compared to the non-CR group (16 out of 137, 12%) (*P* = 0.02). The EQ-5D scores 30 days after discharge were higher in the remote CR group as compared to the outpatient CR group (0.87 ± 0.15 vs. 0.80 ± 0.14, *P* = 0.03), although the EQ-5D scores at discharge were similar for both groups.

**Table 1:** Clinical characteristics of patients hospitalized for HF

	Outpatient CR	Remote CR	non-CR	P
N	69	30	137	
Background				
age, yrs.	59±18	70±9 a	69±20 b	.01
male n, %	42 (61%)	12 (40%) a	56 (41%) b	.02
BMI, %	25±6	23±3	24±6	.36
EF, %	34±7	33±10	38±20	.11
NYHA IV n, %	8 (12%)	4 (13%)	18 (13%)	.95
CAD n, %	9 (13%)	4 (13%)	18 (13%)	1.00
EQ5D score at discharge	0.77±0.11	0.82±0.19	-	.10
Outcome				
EQ5D score 30 days after discharge	0.80±0.14	0.87±0.15 a	-	.03
Emergency readmission n, %	2 (3%)	0 (0%)	16 (12%) bc	.02

Patients’ characteristics were compared using the *χ*^2^ test for non-continuous variables or an unpaired *t* test for continuous variables

*CR* cardiac rehabilitation, *HF* heart failure, *BMI* body mass index, *EF* ejection fraction, *NYHA* New York Heart Association, *CAD* coronary artery disease

^a^*P* < 0.05 between patients with outpatient CR and remote CR

^b^*P* < 0.05 between patients with outpatient CR and non-CR

^c^*P* < 0.05 between patients with remote CR and non-CR

## Discussion

We could successfully demonstrate the improvement in the short-term emergency readmission rate in the remote CR and outpatient CR groups as compared to the non-CR group. The QOL score was rather high in the remote CR group as compared to the outpatient CR group. In Japan, as of May 15, 2020, the emergency readmission for cardiovascular diseases was not influenced by the COVID-19 pandemic. Therefore, we think that the effect of remote CR on emergency readmission could be accurately evaluated in this study.

In 2005, the first large study of remote CR for patients with HF (*n* = 426) was conducted in Europe to compare the clinical outcomes in three groups: the “nurse-telephone support” which was offered once a month, the “home telemonitoring and the nurse-telephone support” wherein the required medical equipment (weighing scale, sphygmomanometer, and electrocardiogram) was supplied to the patient and nurse telephone support was offered in the same manner as in the “nurse-telephone support” group, and the “usual care” by the primary care doctor [3]. The remote management groups showed a better cardiac prognosis than the “usual care” group. The “nurse-telephone support” and “home telemonitoring and nurse-telephone support” groups had the same levels of improved cardiac prognosis, suggesting the importance of nurse-telephone support by itself. In another large-scale remote CR study of patients with HF in the USA (*n* = 1437) [4], the remote CR group received telephone support by a nurse specialized in HF and medical device provision. As a result, the remote CR group had a better short-term prognosis than the standard treatment group. Recently, a large-scale remote CR study (*n* = 1571) was conducted among German patients hospitalized for HF [5]. In this study, patients were provided with medical devices including emergency liaison portable phones, physician-initiated 24-h medical support based on transmitted data, and monthly telephone support to provide HF education. As a result, cardiovascular events were significantly reduced in the remote CR group as compared to the standard care group (hazard ratio 0.70, *P* = 0.028). According to these reports, telephone support by HF nurses and active intervention by physicians can be recognized as essential components for remote CR in patients with HF. In addition, with telephone consultations by CR staff, the appropriate advice and reassurance could be provided to the patients, which is relevant to our findings that the EQ-5D scores 30 days after discharge were high in the remote CR group (Table 1). Concordantly, the dropout rate of the remote CR group was very low (1-7% in the previous studies and 0% in our study), given that the reported dropout rate in outpatient CR ranged from 12 to 82% [8]. Although the outcomes of the remote CR group were compared only with those of the non-CR group in the previous reports [3–5], we could comprehensively compare the outcomes of outpatient CR, remote CR, and non-CR groups in this study.

The initiation of CR in the early phase of hospitalization followed by the patient’s perception of heart failure appears to enable a smooth transition from in-hospital CR to remote CR, reducing the medical staff’s time and effort required for remote CR. In our hospital, the time burden of the medical staff that was managing the remote CR service was 180 min per month, consisting of weekly CR meetings and bi-weekly telephone consultations. Also, there were financial concerns associated with the introduction of remote CR. Basically, medical fees would be higher for remote CR. In addition, many telemedicine devices for patients with HF would be expensive. Our remote CR services amounted to, in total, approximately $3000, including the initial expenses for the production of the video, DVD, and booklet, and telephone charges. Also, the medical devices were owned by each patient, and the number of steps per day was counted by the health app installed in each patient’s mobile phone. Such financial burdens on the patients might affect their participation rate in the remote CR program. This should be resolved for the future promotion of remote CR.

Only 3.8% of medical institutes in Japan could provide remote CR as of April 13, 2020 [9]. Advancements in information technology and telemedicine equipment are expected to promote participation in the remote CR program. Large-scale studies using wearable devices such as Apple Watches have been conducted [10], but their low diagnostic accuracy makes it difficult to apply them into the remote CR of patients with HF. Remote monitoring of blood pressure, electrocardiogram, and oxygen saturation that is easily handled even by the elderly would enable more advanced remote CR. In addition, the problem of data security in remote management must be cleared [5]. Meanwhile, medical safety issues in remote management may also arise, which should also be addressed in order to promote remote CR in the future [11]. The extent to which the healthcare supervisor is responsible for the clinical information detected on remote monitoring (e.g., ventricular tachycardia and ventricular fibrillation) can also pose a legal problem [12]. Each country’s unique healthcare situation and relevant regulation system should be considered.

### Limitations

Because our remote CR program was launched in 2019, the study population in the remote CR group is still small. However, during and after the COVID-19 pandemic, participation in the remote CR program will be increasingly promoted. Further large-scale investigations on long-term prognosis of remote CR are warranted. Considering that the main reason for non-participation in CR programs is the distance between patient residences and the hospital [13], the introduction of the remote CR program could possibly promote participation in CR. However, less-motivated patients might not participate even in remote CR program. At least, EQ-5D scores of anxiety and depression at discharge were not different between the outpatient CR and remote CR groups, which should be further investigated.

## Conclusions

HF patients might be highly susceptible to COVID-19 infection. Therefore, cardiologists and nurses may be hesitant to commence CR for them. However, adequate management of heart diseases, including CR, is still clinically valuable. In order to improve the prognosis of HF patients, we can promote adequate lifestyle guidance and early CR intervention during hospitalization along with remote CR services with telephone support after discharge.

## Data Availability

The datasets used and analyzed in the presented study are available from the corresponding author on reasonable request.

## Abbreviations

CR: Cardiac rehabilitation
HF: Heart failure
EF: Ejection fraction
ED: Emergency declaration
NYHA: New York Heart Association
DVDs: Digital video disks
QOL: Quality of life
